# tiny-count: a counting tool for hierarchical classification and quantification of small RNA-seq reads with single-nucleotide precision

**DOI:** 10.1093/bioadv/vbad065

**Published:** 2023-05-18

**Authors:** Alex J Tate, Kristen C Brown, Taiowa A Montgomery

**Affiliations:** Department of Biology, Colorado State University, Fort Collins, CO 80523, USA; Department of Biology, Colorado State University, Fort Collins, CO 80523, USA; Cell and Molecular Biology Program, Colorado State University, Fort Collins, CO 80523, USA; Department of Biology, Colorado State University, Fort Collins, CO 80523, USA; Cell and Molecular Biology Program, Colorado State University, Fort Collins, CO 80523, USA

## Abstract

**Summary:**

tiny-count is a highly flexible counting tool that allows for hierarchical classification and quantification of small RNA reads from high-throughput sequencing data. Selection rules can be used to filter reads by 5′ nucleotide, length, position of alignments in relation to reference features, and by the number of mismatches to reference sequences. tiny-count can quantify reads aligned to a genome or directly to small RNA or transcript sequences. With tiny-count, users can quantify a single class of small RNAs or multiple classes in parallel. tiny-count can resolve distinct classes of small RNAs, for example, piRNAs and siRNAs, produced from the same locus. It can distinguish small RNA variants, such as miRNAs and isomiRs, with single-nucleotide precision. tRNA, rRNA, and other RNA fragments can also be quantified. tiny-count can be run alone or as part of tinyRNA, a workflow that provides a basic all-in-one command line-based solution for small RNA-seq data analysis, with documentation and statistics generated at each step for accurate and reproducible results.

**Availability and implementation:**

tiny-count and other tinyRNA tools are implemented in Python, C++, Cython, and R, and the workflow is coordinated with CWL. tiny-count and tinyRNA are free and open-source software distributed under the GPLv3 license. tiny-count can be installed via Bioconda (https://anaconda.org/bioconda/tiny-count) and both tiny-count and tinyRNA documentation and software downloads are available at https://github.com/MontgomeryLab/tinyRNA. Reference data, including genome and feature information, for certain species can be found at https://www.MontgomeryLab.org.

## 1 Introduction

Small RNAs interact with larger RNAs through base-pair interactions to direct mRNA decay or translational repression, or to promote RNA, DNA, or histone modifications. There is tremendous diversity among small RNAs both between and within species. The canonical classes of small RNAs—microRNAs (miRNAs), piwi-interacting RNAs (piRNAs), and small interfering RNAs (siRNAs)—are unified by their association with Argonaute/Piwi proteins (Ghildiyal and Zamore, 2009). These classes of small RNAs can be distinguished by their genetic requirements, length, 5′ nucleotide (nt), and precursor structure (Farazi *et al.*, 2008). Numerous other classes of short RNAs, such as tRNA and rRNA fragments, also play diverse roles in gene regulation (Aalto and Pasquinelli, 2012).

High-throughput sequencing is a widely used tool for discovery and analysis of small RNAs (Veneziano *et al.*, 2016). The diversity and complexity of small RNA pathways present several computational challenges when classifying and analyzing the reads generated by high-throughput sequencing. For example, small RNA libraries are often contaminated with decay intermediates of longer RNAs, such as rRNAs and mRNAs, that can also produce functional small RNAs (Lambert *et al.*, 2019). These contaminants can be difficult to distinguish from authentic small RNAs produced from the same features. Additionally, differential or imprecise processing can generate functionally distinct small RNAs, such as miRNA variants called isomiRs, that differ by as little as 1 nucleotide (Bartel, 2018). Further complicating data analysis, distinct classes of small RNAs, such as piRNAs and siRNAs, can be produced from the same locus (Das *et al.*, 2008; Kawamura *et al.*, 2008). The tinyRNA project addresses these challenges with tiny-count, a single-nucleotide precision counting tool designed to quantify small RNAs and other short RNAs from high-throughput sequencing data.

## 2 Methods and results

### 2.1 Overview

tiny-count evaluates sequence alignments for feature assignment utilizing the genomic array and GFF reader from HTSeq (Anders *et al.*, 2015). User-defined selection rules can be used to classify reads based on their sequence attributes, which include 5′ nt and length, as well as their relationship to features of interest, such as strandedness, positional overlap, and number of mismatches. Selection rules can require exact, partial, nested, or anchored alignment of reads to features of interest. Alignment can be relative to the annotated 5′ or 3′ ends of features of interest or shifted by any number of nucleotides specified by the user. Each selection rule must specify a hierarchy value, and rule sets with heterogeneous hierarchy values can be used to preferentially assign reads to one class over another. Reads can also be subclassified for individual features based on these rules. tiny-count will distinguish and quantify reads for every rule simultaneously while preserving information for each feature-rule combination for subsequent analysis. Hence, the user can perform downstream analysis treating subsets of small RNAs produced from the same locus as distinct features.

For each sequence, counts are optionally normalized by the number of genomic alignments and/or by the number of features the sequence matched. tiny-count can also normalize by library size using the common small RNA normalization method, reads per million mapped reads (rpm). Alternatively, users can specify a custom normalization factor for each library, thereby enabling the use of spike-in counts or other metrics for normalization. tiny-count produces a CSV-formatted table with counts for each feature that are optionally subclassified based on the user-defined selection rules. Additionally, the total counts for each rule or class of small RNAs are reported, as are summary statistics and 5′ nt and length distribution tables.

tiny-count can be installed via Bioconda or downloaded from GitHub. To run tiny-count as a stand-alone program, the user provides SAM files with read alignment information for each library. The user can provide a GFF file containing positional information for features of interest. In the absence of a GFF file, each unique reference sequence is treated as a distinct feature. This allows for analysis of data from alignments directly to features of interest rather than to a genome. Read selection rules and sample information are specified in CSV files, and paths to these files and other run information are specified within a YAML-formatted configuration file. Alternatively, tiny-count can be run as part of the tinyRNA end-to-end data analysis workflow described below.

### 2.2 Precision counting

**Fig. 1.**
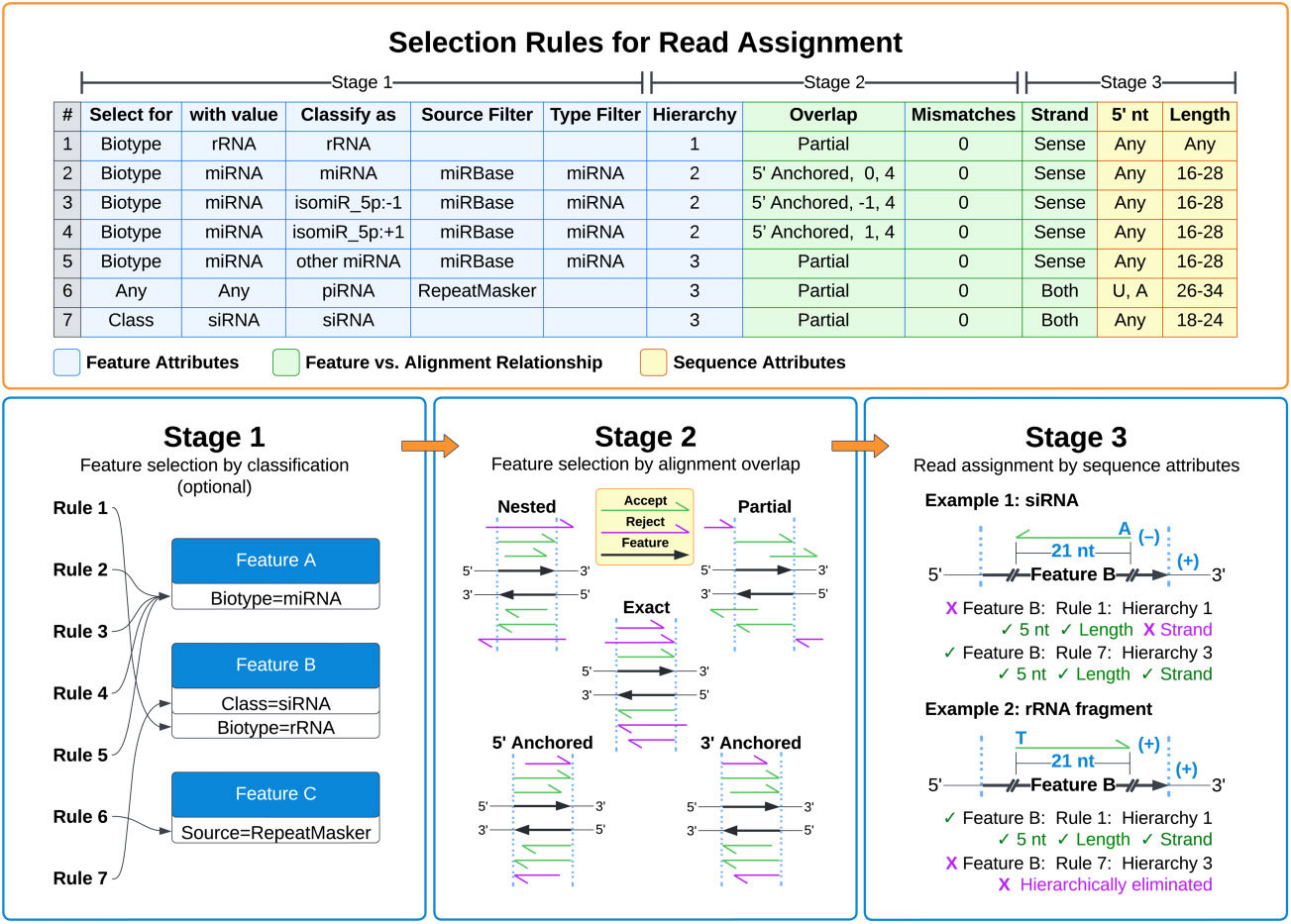
The tiny-count selection scheme. Small RNA read and alignment attributes are specified within a CSV-formatted file in which each row corresponds to a selection rule. The Stage 1 selection attributes reflect the variety of sources used for small RNA annotation and classification. Feature selection and read filtering occurs in three stages. Stage 1: information about features specified by the user is extracted from a GFF file. If a GFF file is not provided, this step is omitted. Stage 2: features are selected based on their overlap with read alignments and edit distance (mismatches) to the reference sequence. Stage 3: reads are assigned to features passing Stages 1 and 2 selection based on their sequence attributes, such as 5′ nt and length, as well as their strand relationship to the feature. Reads meeting all selection criteria for a specific rule are then assigned to features. If there is more than one matching feature, reads are evenly distributed or preferentially assigned to one over the others based on user-defined hierarchy values. In Example 1, a hypothetical siRNA read is incompatible with classification as an rRNA as defined in Rule 1 because of strand incompatibility but is compatible with classification as an siRNA as defined in Rule 7. Conversely, in Example 2, a hypothetical rRNA fragment read is compatible with both Rules 1 and 7 but is assigned to Rule 1 because of its lower hierarchy value

tiny-count provides precise control over the attribution of reads at alignment loci with user-defined selection rules. Any number of selection rules can be defined to categorize reads. The selection process resolves ambiguities in feature assignment at loci with multiple overlapping features and the associated loss of counting precision. It also allows for reads from distinct classes of small RNAs produced from the same feature or genomic interval to be treated separately during counting and analysis. Feature selection and read matching occurs in three stages (Fig. 1). In Stage 1, features of interest are retrieved from GFF-formatted input files based on user-specified attributes, including source (listed in column 2 of the GFF), type (listed in column 3 of the GFF), and annotation (listed in column 9 of the GFF). If reads are counted from alignments directly to sequences of interest, rather than to a genome containing feature intervals of interest, then both the GFF file and Stage 1 can be omitted.

During Stage 2, reads are assessed for their positional overlap with the features that were selected in Stage 1. If a GFF file is not provided, reads are instead evaluated for their overlap with each reference sequence, as specified in the header section of the SAM-formatted alignment file. Selection rules can specify partial, nested, or exact overlap with features of interest, with the option to shift the annotated coordinates by any number of nucleotides in the 5′ or 3′ direction. Additionally, features can be selected based on whether their 5′ or 3′ ends are anchored to the alignment or to a user-defined position relative to the alignment. The number of mismatches relative to features of interests can also be specified if such mismatches were permissible at the alignment step. This can be a range or a single value, which can be used to capture sequence variants. Features passing the selection criteria in Stage 2 are sorted by user-defined values for hierarchical selection in Stage 3.

In Stage 3, features are matched with reads based on each read’s attributes, such as its 5′ nt, length, and strandedness relative to the feature. Reads are assigned at Stage 3 to matching features with the lowest hierarchy value that meet all selection criteria and are thus excluded from any other overlapping features at the alignment locus with higher hierarchy values. Features which pass the selection process are assigned all counts or a normalized portion of the counts associated with the alignment sequence. By default, a sequence with n read counts and m genomic alignments is assigned (n/m)/k reads, where k is the number of features that pass selection at the locus. The m and k normalization factors are both optional.

The hierarchical selection scheme utilized by tiny-count allows users to assign reads preferentially or uniformly to features of interest. For example, it is often necessary to filter out reads that align to structural RNAs. Using tiny-count, reads that align to rRNAs or other abundant structural RNAs can be excluded from assignment to other features by giving the structural RNA classes lower hierarchy values (closer to or equal to 1). In this approach, counts from structural RNAs are excluded from assignment to other classes of small RNAs and are reported separately, which can provide an important indicator of RNA integrity or could point to biological relevance of such reads (Lambert *et al.*, 2019).

### 2.3 Applications

tiny-count is designed to provide the highest level of user control over read selection and classification when quantifying high-throughput sequencing data. A few possible applications are described below to highlight potential usage of tiny-count, although we anticipate its functionality extending far beyond these examples.

**miRNAs and isomiRs:** The functionality of tiny-count can be illustrated by considering miRNAs, for which some variation at the 3′ end is often permissible but variation at the 5′ end would shift the seed sequence and alter target recognition (Bartel, 2009). Pooling counts for all sequences that overlap with a miRNA locus can produce inaccurate results, whereas limiting the analysis to only reads with exact matches to annotated miRNAs may undercount relevant data. We commonly use a rule for miRNAs that captures reads with nested overlap within annotated miRNA loci. We require an exact match with the annotated 5′ ends of miRNAs and allow extensions up to 4 nt beyond the annotated 3′ ends to account for variation in 3′ end processing (Overlap = 5′ anchored, 0, 4; see Selection Rules example in Fig. 1). miRNAs are typically 20–24 nt long and we often expand this range to account for outliers (Length = 16–28). There is typically no reason to restrict the 5′ nt because this will be dictated by the annotated 5′ ends of the miRNAs (5′ nt = Any). To identify miRNA isoforms (isomiRs) for which the 5′ ends are shifted upstream by 1 nt, an additional rule could be added in which the overlap parameter is offset from the 5′ ends by −1 relative to the annotated coordinates (Overlap = 5′ anchored, −1, 4). Another rule could be defined to capture 5′ isoforms shifted downstream by 1 nt (Overlap = 5′ anchored, 1, 4) (Fig. 1). If desired, additional rules could be defined to distinguish other possible isomiRs, or a catch-all rule could be specified to capture all other partially overlapping reads (Overlap = Partial). By assigning a higher hierarchy value to this catch-all rule, only reads that are not assigned to the other miRNA rules would be counted. There is no need to distinguish the hierarchies of the other miRNA rules since they are mutually exclusive of each other due to distinct 5′ offsets of the overlap selectors (i.e. 0, −1, and 1). Alternatively, if the user wanted to capture reads that overlap exactly with annotated miRNAs, an exact overlap could be specified (Overlap = Exact). Regardless of how the rules are defined, the counts for each miRNA-rule pair will be listed as separate features in the counts table.

**Promoter-derived small RNAs:** Small RNAs are often produced from promoter regions of coding genes (Kasschau *et al.*, 2007; Zamudio *et al.*, 2014). To capture small RNA reads derived from promoter regions, which are often imprecisely annotated, a user could first define a selection rule to capture reads from annotated gene bodies (e.g. Overlap = Nested, 0, 0; Hierarchy = 1). Then in a second rule, the user could specify nested overlap within a region shifted by some number of nucleotides upstream of gene bodies and assign it a higher hierarchy value (e.g. Overlap = Nested, −1000, 0; Hierarchy = 2). The first rule would capture all reads nested within gene bodies, whereas the second rule would capture reads not aligned to gene bodies in the first rule (because of its hierarchy value) but nested within some number of nucleotides upstream (1000 in this example). Due to variability in promoter lengths, the user could specify any number of distinct bins upstream of the transcribed region to provide greater resolution.

**Nematode 22G- and 26G-RNAs:** In *Caenorhabditis elegans* and other nematodes, many genes produce two classes of small RNAs with distinct genetic requirements, one of which is characterized by sequence lengths of ∼22-nt (22G-RNAs) and the other by ∼26-nt species (26G-RNAs). Both classes are comprised primarily of sequences with a 5′G that are produced antisense to their transcripts of origin (Billi *et al.*, 2014). These two classes can be distinguished with tiny-count by defining two selection rules, one that captures reads with length 22-nt (Length = 22) and one that captures reads with length 26-nt (Length = 26), and both of which include only 5′G-containing antisense reads (5′ nt = G; Strand = Antisense). There is no need to distinguish the hierarchy in this example because reads can only match one rule or the other due to the length restrictions. Because of variation in processing, the lengths and 5′ nt could be assigned a more permissive set of values to capture additional reads, although this would come at the expense of categorical resolution.

### 2.4 The tinyRNA workflow

**Fig. 2.**
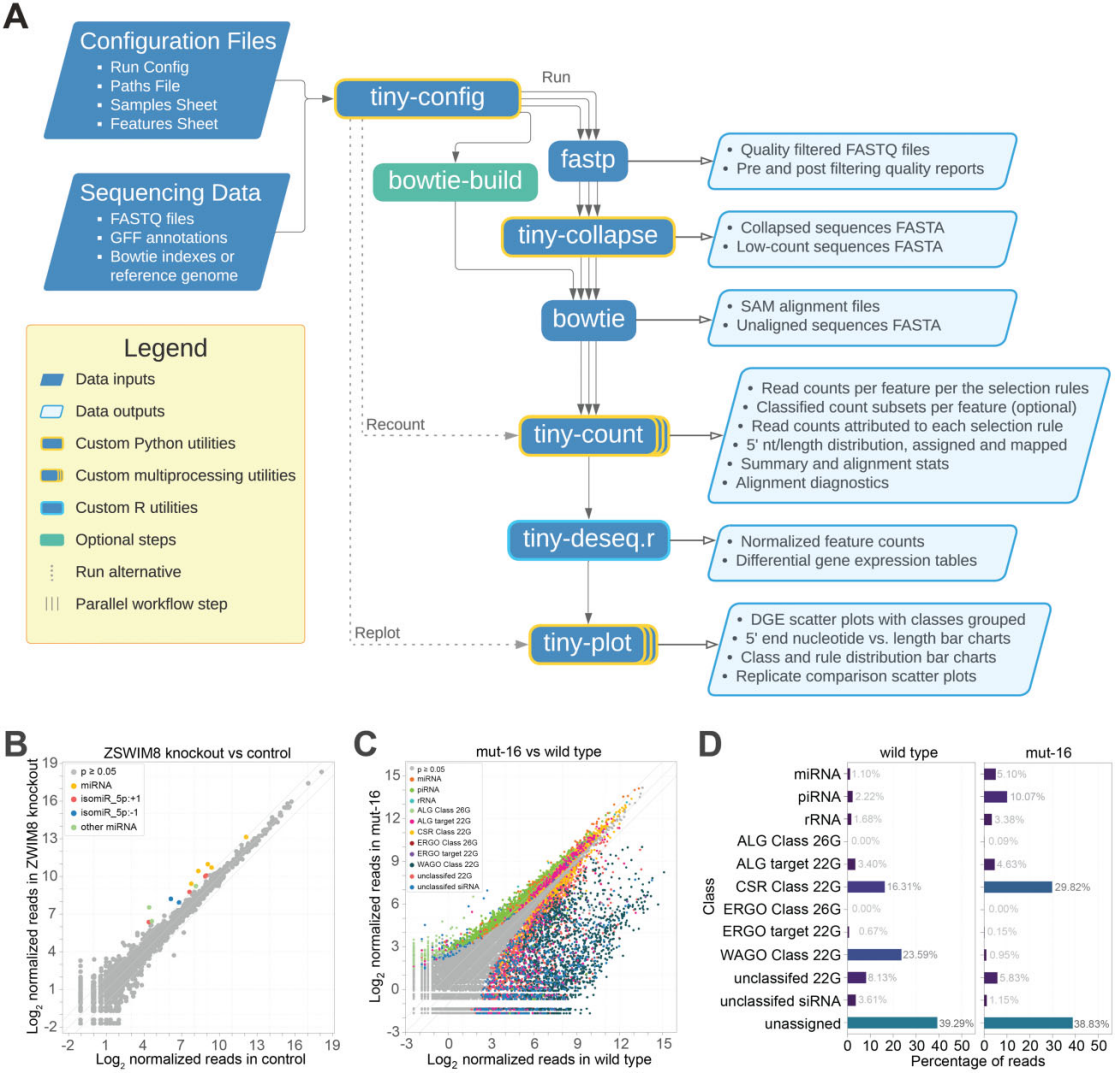
The tinyRNA workflow. (**A**) tinyRNA flowchart. A selection of graphical outputs from a human miRNA analysis (Shi *et al.*, 2020) (**B**), and a *C. elegans* total small RNA analysis (Reed *et al.*, 2020) (**C** and **D**). Note that some post-run editing of the plots was done due to space constraints. The outputs of each tinyRNA run, including quality reports, processed data, mapping and assignment statistics, counts tables, size plots, class charts and a variety of scatter plots, are placed in a timestamped directory with full run documentation

tiny-count can be run as a stand-alone program or as part of the tinyRNA small RNA data analysis workflow (Fig. 2A). When running tinyRNA, execution begins with the automated generation of a workflow in Common Workflow Language (CWL) (Crusoe *et al.*, 2022). CWL is portable, scalable across different computing resources, and, like the tinyRNA project, its development is community driven. Preprocessing of FASTQ files, including adapter trimming and quality filtering, is performed with fastp (Chen *et al.*, 2018). Unique sequences are counted and collapsed by the tinyRNA collapser tool, tiny-collapse, substantially reducing the resource demands of genomic alignment and feature counting. tiny-collapse can also trim the degenerate bases often included in the adapter sequences used in library preparation. Collapsed reads are aligned to a reference genome using Bowtie allowing 0–3 mismatches (default = 0, specified within the run configuration file) (Langmead *et al.*, 2009). tiny-count is then used for read quantification, as described above. If the data contain biological replicates, differential expression analysis is automatically performed by tiny-deseq, a wrapper around DESeq2 (Love *et al.*, 2014), using the counts tables generated by tiny-count. Finally, the outputs of tiny-count and tiny-deseq are used to produce publication-ready plots with the tinyRNA plotter tool, tiny-plot.

tinyRNA provides a simple command-line-based solution for integrating precision classification and analysis of short RNAs into a general automated data analysis pipeline. To the best of our knowledge, tinyRNA adds a higher degree of precision and user control when processing, counting, and classifying reads than other software suites. However, tinyRNA lacks the specialized functionality found in some tools, such as integration of different data types, querying of public data repositories, and automated classification of small RNAs (Han *et al.*, 2015; Li *et al.*, 2020; Rueda *et al.*, 2015; Stocks *et al.*, 2018; Wu *et al.*, 2017). With tinyRNA, users must provide all reference data for their analysis. This approach allows users to use their preferred reference sequences and to analyze any set of features regardless of availability in a publicly accessible database. It also avoids challenges associated with querying online databases from some servers or when working offline. Care should be taken by the user to ensure that reference data is from a reliable and up-to-date source. This is particularly important for small RNAs, which are often absent or poorly annotated in GFFs distributed through non-specialized databases.

Despite being a command-line tool, very little terminal software experience is necessary to run tinyRNA because input files and parameters are passed to the workflow via text-based configuration files. After preparing the input files, the entire tinyRNA workflow can be run with a single command, *tiny run --config run_config.yml*. Alternatively, certain steps of the workflow, such as counting and plotting, can be repeated on a completed run if the user chooses to modify the parameters, such as the selection rules or plot appearance.

We tested tiny-count and the tinyRNA workflow on human cell line datasets by quantifying miRNAs in control and ZSWIM8 knockout lines (Fig. 2B) (Shi *et al.*, 2020). We also tested their ability to accurately quantify multiple classes of small RNAs in parallel using *C. elegans* germline tissue datasets from wild type and *mut-16* mutant animals (Fig. 2C and D) (Reed *et al.*, 2020). In each of these tests, tinyRNA provided results that were consistent with the original published analyses but with greater resolution of affected features. For example, in the human dataset, tinyRNA identified several isomiRs that were upregulated in ZSWIM8 knockout cell lines that were not previously reported (Fig. 2B). In the *C. elegans* dataset, tinyRNA identified numerous unclassified siRNAs that were depleted in *mut-16* mutants and distinguished between affected 22G- and 26G-RNAs produced from the same genes (Fig. 2C).

### 2.5 Performance

To our knowledge, tiny-count is the only read counting program that combines hierarchical assignment of reads to user-defined categories, selection based on small RNA length and 5′ nt, 5′- or 3′-anchored alignments, and single nucleotide control over alignment overlap (Table 1). Nor to our knowledge is the user-defined small RNA counting and classification scheme of tiny-count available in small RNA pipeline software other than tinyRNA. tiny-count’s unique features enable the highest level of accuracy in counting and classifying small RNAs. Because of tiny-count’s hierarchical read assignment strategy, data does not have to be prefiltered or sorted before counting, and multiple classes of small RNAs can be analyzed in parallel. tiny-count is faster than HTSeq-count, but it is slower than featureCounts, which is an exceptionally fast general purpose counting program that is not readily configurable for precision counting of diverse small RNA classes (Table 1) (Anders *et al.*, 2015; Liao *et al.*, 2014). Furthermore, tiny-count, but not HTSeq-count nor featureCounts, can extract read counts from collapsed SAM files (i.e. reads are collapsed and tallied within each sequence record to minimize the number of genome alignments required). When SAM files containing collapsed reads are used in tiny-count, its performance begins to approach that of featureCounts while allowing for quantification of multiple classes in parallel (45.1 versus 2.4 s) (Table 1). Currently, tiny-count can only accommodate SAM files containing collapsed read data if the reads were collapsed with FASTX-Toolkit, tiny-collapse, or custom programs that embed the counts within read identifiers formatted similarly to either of these tools.

**Table 1.** Comparison of read counting programs applied to small RNA-sequencing data

Read counting program	Strand selectivity	Length selectivity	5′ nt selectivity	Hierarchical assignment	5′ or 3′ anchoring^a^	Runtime^b^
HTSeq-count	Yes	No	No	No	No	12 m 57 s
featureCounts	Yes	Yes	No	No	No	2.4 s
tiny-count HTSeq StepVector	Yes	Yes	Yes	Yes	Yes	3 m 44 s
tiny-count Cython StepVector	Yes	Yes	Yes	Yes	Yes	2 m 35 s
tiny-count Cython StepVector with collapsed reads^c^	Yes	Yes	Yes	Yes	Yes	45.1 s

a Reads can be selected by 5′- or 3′-end alignment to 5′- or 3′-end of feature or shifted by any number of nucleotides.

b Approximately 13 million genome alignments and ∼50k features with an assignment rate of ∼65%. Benchmarks were taken on an HPE ProLiant server using one Xeon E5-4640V4 2.10 GHz CPU and allowing up to 256 GB memory usage.

c Reads collapsed prior to mapping. Tallied read counts are contained within SAM records and captured by tiny-count.

Most steps in tiny-count and tinyRNA run concurrently across libraries to minimize runtime. A user-defined global thread count is set for fastp and Bowtie to utilize multi-core computing resources. Performance-critical sections of tiny-count have been carefully written with respect to the resulting bytecode, or internal representation of the program in the CPython interpreter, to optimize execution. Additionally, we developed a custom implementation of the HTSeq StepVector written in Cython, a C language extension to Python, to address inefficiencies in iteration and intermediate object creation. The StepVector is a data structure which represents piecewise constant values at low resolution and is used by tiny-count to resolve features that overlap a given interval by at least one nucleotide. This data structure is heavily utilized during read assignment and because of our implementation, overall runtimes in tiny-count have been reduced by an average of ∼30% relative to an implementation using the HTSeq StepVector (Table 1).

### 2.6 Convenience and reproducibility

tiny-count and the tinyRNA workflow are designed to be operable by users with minimal command-line computing skills and little or no small RNA data analysis experience. Configuration parameters for the individual tools within tinyRNA are contained within modules rather than residing in a single monolithic file. This allows the tinyRNA pipeline to be executed with a single command whose sole argument is the primary configuration file. Command-line parameters for each step in the tinyRNA pipeline can be modified by editing the primary configuration file. Thus, the user does not need to be familiar with the various tools used by tinyRNA, such as fastp and Bowtie, to utilize their extensive functionality. A standard small RNA analysis configuration that follows best practices in the field is provided as a YAML-formatted template. Upon tinyRNA run completion, copies of configuration files are placed in timestamped output directories that are organized into subdirectories by step to simplify documentation and collaboration. Most tinyRNA components are written in Python using object-oriented design patterns for transparency and easy modification by advanced users. The tinyRNA pipeline can be extended to include any number of additional steps by modifying the workflow CWL. These advanced modifications are permissible by the installation script for easy integration into a custom workflow.

## 3 Summary

tiny-count performs precision counting of small RNAs from high-throughput sequencing data. Utilizing tiny-count, the tinyRNA workflow enables end-to-end analysis of small RNA high-throughput sequencing data following best practices, producing complete run documentation for accuracy and reproducibility. It is compatible with data from any species containing reference sequences for the genome or small RNAs of interest. tiny-count and tinyRNA can accommodate relatively simple data analyses, such as miRNA expression in mammalian cell lines, to complex analyses of multiple classes of small RNAs from whole organisms, such as *Arabidopsis*, *Drosophila*, and *C. elegans.*

## Data Availability

There are no new data associated with this article.
